# Laser Processing of Transparent Wafers with a AlGaN/GaN Heterostructures and High-Electron Mobility Devices on a Backside

**DOI:** 10.3390/mi12040407

**Published:** 2021-04-06

**Authors:** Simonas Indrišiūnas, Evaldas Svirplys, Justinas Jorudas, Irmantas Kašalynas

**Affiliations:** 1 Laser Microfabrication Laboratory, Center for Physical Sciences and Technology (FTMC), Savanoriu Ave. 231, LT-02300 Vilnius, Lithuania; evaldas.svirplys@ftmc.lt; 2 Terahertz Photonics Laboratory, Center for Physical Sciences and Technology (FTMC), Saulėtekio 3, LT-10257 Vilnius, Lithuania; justinas.jorudas@ftmc.lt (J.J.); irmantas.kasalynas@ftmc.lt (I.K.)

**Keywords:** laser micromachining, sapphire, silicon carbide, AlGaN/GaN heterostructures, high-electron mobility devices

## Abstract

Sapphire and silicon carbide substrates are used for growth of the III-N group heterostructures to obtain the electronic devices for high power and high frequency applications. Laser micromachining of deep channels in the frontside of the transparent wafers followed by mechanical cleavage along the ablated trench is a useful method for partitioning of such substrates after the development of the electronics on a backside. However, in some cases damage to the component performance occurs. Therefore, the influence of various parameters of the laser processing, such as fluence in the spot size, substrate thickness, orientation, and the polarization of focused laser beam, to the formation of damage zones at both sides of the transparent substrate with thin coatings when ablating the trenches from one side was investigated. The vicinity effect of the ablated trenches on the performance of the electronics was also evaluated, confirming the laser micromachining suitability for the dicing of transparent wafers with high accuracy and flexibility.

## 1. Introduction

In high power, high temperature resistance, high frequency electronics applications wide bandgap III-N group semiconductors (nitrides) have an advantage over conventional silicon electronics [1]. The III-N group heterostructure layers are usually grown on a wide-bandgap (transparent for visible light) materials, such as sapphire, silicon carbide (SiC), or gallium nitride (GaN) which provide small or even no lattice mismatch for the epitaxial layers in order to obtain superior high power electronic devices based on two-dimensional electron gas (2DEG) with high-electron mobility [2,3]. After the growth of required heterostructure layers on the substrate and manufacturing of the electronics, the wafer has to be partitioned into smaller pieces which contain separate electronic components and circuits. For that, mechanical scribing and cleavage or dicing with a diamond saw are widely used in industry providing relatively fast and cheap solutions, however, applicability of these tools for accurate and flexible shape partitioning of closely situated (distance < 100 µm) electrical components is challenging or even impossible. Laser micromachining of transparent materials can be invoked for accurate processing since the positioning accuracy and the size of ablation zone as small as several tens of micrometers to several micrometers can be achieved [4].

Having in mind that the substrate side with the electrical components should be hindered as little as possible, the ablation of deep channels in the backside of the substrate by direct laser ablation (DLA) followed by mechanical cleavage along the ablated trench line has been proposed for partitioning the samples while avoiding the damage or contamination to the front side [5]. However, in this work we observed that in some cases this method may result in the formation of the damage zones on both sides of the wafer near the ablated trench line, in the material layers on the “good” side of the substrate. Since backside damage formation is undesirable effect of DLA, it may act as a limiting factor for a wider adoption of DLA methods hindering the application of laser scribing for the separation of electronics components on the transparent wafers. Thus, the investigation of various operation regimes under destructive light mater interaction is crucial for processing of transparent materials exploiting all advantages of the laser micromachining.

Damage phenomena were reported in the literature, regarding laser cutting of thin sheets of transparent materials (glasses, fused silica) but without the electronic devices on a backside. In particular, it was demonstrated that some damage to the rear side and volume of the glass slab was made during the laser scribing process [6]. The damage on the rear side of the Shott glass substrate was attributed to the laser radiation, escaping from the ablation channel by refraction from the crater walls. In this case damage to the rear side of the substrate can be avoided or significantly reduced by selecting S polarization (polarization vector parallel to the laser scribing direction) which at oblique propagation angle has a higher reflectance from the air-glass interface, compared to P polarization, resulting in the peak fluence of the refracted radiation too low to reach glass damage threshold.

In [7], band-like damage was observed on the backside of laser cut 100 µm-thick aluminum-borosilicate-glass. It was reported that the damage was observed for both S and P polarizations, but was more pronounced in the case of S polarization (polarization vector parallel to the cut line).

Some authors [8] attribute backside damage to the collision of laser-induced plasma-generated stress waves: collision of longitudinal (compression) wave and Rayleigh surface wave, excited on the back surface by the transverse (distortion) stress wave. In this case, it was reported that when ablating the channel in 156 µm-thick borosilicate glass two scans (producing 20–30 µm depth channel) was enough to produce the damage lines on the back surface. With increasing scan number, the depth of the channel increased and the damage lines were appearing closer to the channel plane. The 117 µm distance from the channel to the damage zone was reported. In this case, 150 fs pulses at 800 nm wavelength, 50 µJ pulse energy, and 1 kHz repetition rate were used.

In [9], pump-probe experiments of laser irradiated glass, supplemented by simulations using linear beam propagation method, demonstrated that when ablated crater reaches a certain depth, part of the irradiation energy is deposited in the relatively narrow regions extending from crater sidewalls to the glass volume, at an angle (approximately 24°) to the glass surface. In this case, 80 fs, 47 µJ, 1 Hz, 800 nm laser irradiation was used.

In [10], the distance from the channel to the damage zone in a 90 µm-thick fused silica sheet was reported as 40.6 µm. The rear damage mechanism was explained as local intensity enhancements, caused by the interference of the laser radiation transmitted and reflected in the glass/air interface on the backside of the sheet. Laser irradiation reaches the back surface by refraction from the ablated crater wall and propagation to the rear interface of the sheet. The irregularity of the damage zone was explained by local modifications of the refractive index near the ablated crater. It was reported that P polarized laser beam produces a weaker damage zone due to the low reflectivity of the P polarization at the fused silica/air interface (compared to S polarization). It was proposed to put the fused silica sheet in the distilled water during the laser processing thus further reducing the reflectance at the glass/air interface.

In [11], 10 ps laser pulses were used to ablate Corning Eagle XG and Gorilla glasses. In this case, several types of damage to the rear side of the glass plate were observed. At low scan numbers, circular damage areas with a diameter similar to the focused laser beam diameter, which can be attributed to the diffraction pattern from the laser, formed opaque zone on the front side of the glass plate. At higher (several hundred) scan numbers line-like damage zones on the rear side of the sample at both sides of the cut were manifesting. The distance from the cut line (for 0.7 mm-thick sample) varied with the scan number from 170 µm to 500 µm. At large scan numbers, several distinct damage lines could be observed.

In [12], ablation of channels in fused silica were compared with theoretical model and provided quite good agreement. It was suggested that laser radiation experiences interference in the material surrounding the ablation channel due to interference of radiation refracted from the channel walls, reflected and refracted from the channel walls, and entering the material from the region surrounding the crater, where fluence is too low for nonlinear absorption. Spike-like damage regions emanating at an angle to the crater walls were observed experimentally and also replicated in the model as regions of high free electron density.

The aim of this paper was to investigate the formation of damage zones in the coating, deposited on the backside of the transparent substrate, during the laser scribing, using various laser processing parameters. Formation of damage zones in backside coatings in a broad range of various thickness substrates was performed. A method to reduce the area of the damage zones by oblique laser beam scanning was presented and validated by laser-machining of SiC wafers with real AlGaN/GaN heterostructures and high-electron mobility devices on a backside.

The effect of laser beam damage to the surface morphology and the performance of real electronic devices made on GaN/AlGaN heterostructures, grown on the back side of the sapphire or SiC substrates [13], was investigated. Additional research was also performed on the transparent glass (soda-lime) substrates with a thin gold film deposited on the back side, to avoid unnecessarily high costs of material.

## 2. Experimental Setup

The samples were the 350–500 µm-thick SiC and sapphire wafers with developed semiconductor layers and metal contacts and the 0.15–4.4 mm thick soda-lime glasses with a gold layer of 30 nm thickness, deposited on one of the surfaces by a DC sputter coater Q150T ES (Quorum Technologies). The SiC and sapphire samples under processing were attached to the glass plate with an optical cleaning tissue in between, trying to avoid scratching of the semiconductor layers and metal contacts. Soda-lime samples were processed using a special holder so that the processed zones had no physical contact with any substance on both sides (sample was hanging in the air).

**Figure 1:**
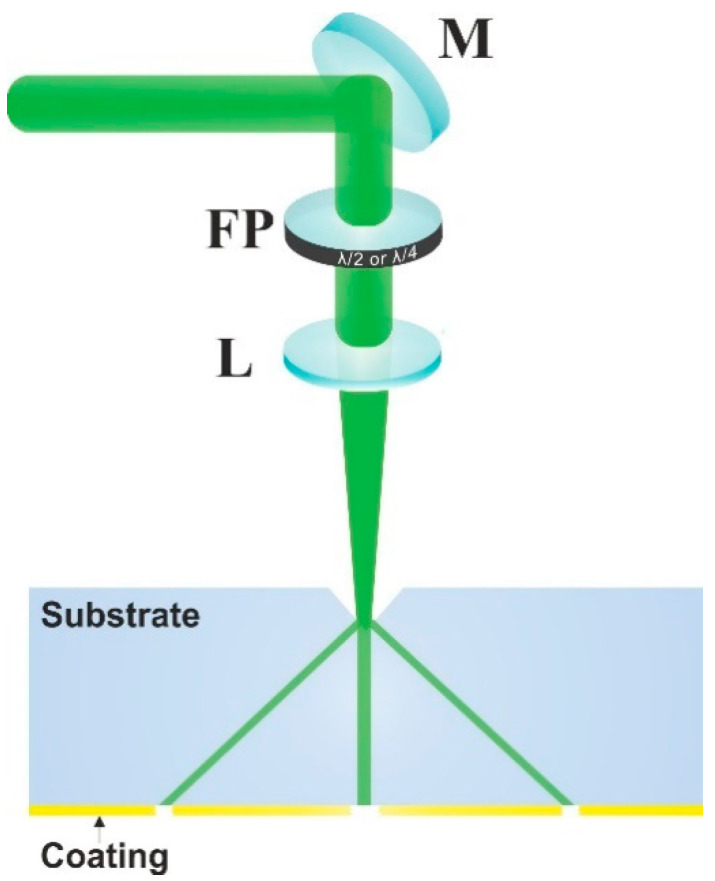
Experimental setup. M—mirror, FP—phase plate, L—focusing lens.

The experiments were conducted using picosecond laser Atlantic (Ekspla): fundamental harmonics wavelength 1064 nm, pulse energy up to 150 µJ, pulse repetition rate up to 1 MHz, pulse duration 10 ps, second (532 nm) and third (355 nm) harmonics available. A laser beam was focused using one of the focusing 50 mm or 100 mm focal length lenses for 355 nm, and a 50 mm focal length focusing lens for 1064 and 532 nm. Spot size radii using 1064 nm and 532 nm wavelengths were 19.5 µm and 12.5 µm, respectively. A laser beam was scanned by displacing the sample with respect to the laser beam using XYZ translation stages (ALS10020, Aerotech). Scanning speed of 10 mm/s and 100 kHz pulse repetition rate was used for all scribes in the gold coated soda-lime samples. Scanning speed of 100 mm/s and 400 kHz pulse repetition rate using the third harmonics was employed for trench ablation in the SiC and sapphire wafers. Polarization was controlled using an appropriate half wave or quarter wave plate (see Figure 1).

The cross-section of the ablation channel was investigated by performing a perpendicular scribe from the backside and breaking the sample along this scribe. After breaking, the samples were cleaned with a deionized water in an ultrasonic bath.

Channel shape in soda-lime glass and damage to the layers deposited on the backside of various transparent substrates were investigated using an optical microscope.

## 3. Results and Discussion

### 3.1. Laser Scribing of SiC and Sapphire Wafers with Electronic Devices on a Backside

**Figure 2:**
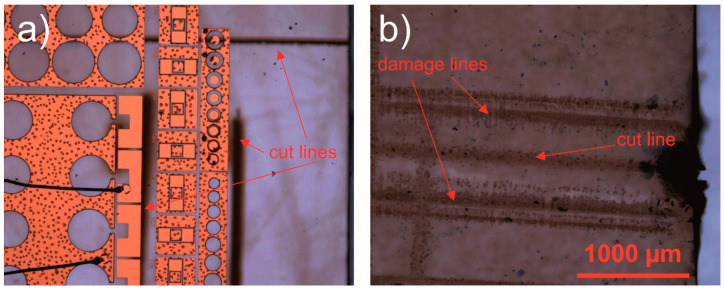
Laser scribe trenches in (**a**) SiC and (**b**) sapphire substrates with semiconductor and metal layers on the backside. Laser cutting was performed from the substrate side. In (**a**), the lenses with the focal length of 50 mm and 100 mm were used to process vertical and horizontal trench lines, respectively. In (**b**), 100 mm focusing lens was used. Formation of the damage lines was observed on both sides of the wafer along the trench but on a sapphire substrate only.

Figure 2 shows optical microscope images of laser ablated trenches in SiC and sapphire substrates, which have AlGaN/GaN heterostructures with electronic devices on a backside. Ablation of trenches was performed always from the substrate side. Damaged areas on the opposite side of the substrate near the cut line were found in the sample shown in Figure 2b only.

### 3.2. Point Damages

**Figure 3:**
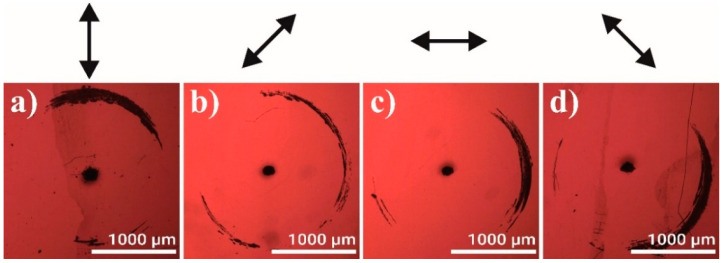
Damage in the 30 nm gold coating on the backside of the 1 mm thick glass plate for various polarization angles, indicated as black arrows above the images (**a**–**d**). The approximate polarization direction is illustrated by the arrows above each image. Laser wavelength 1064 nm, fluence 16 J/cm^2^, 1000 pulses per spot. Measurement labels are in micrometers.

The properties of the damage generation were investigated in detail, employing the 1 mm thick glass samples with a 30 nm thick gold films deposited on the backside. Samples were irradiated by a focused laser beam for some duration without moving the beam or the sample. Figure 3 shows typical damage shapes in a gold coating for several polarization states. The damage areas formed at 800–1050 µm distance from the irradiation spot (black dot in the image center). It was seen that the damage zones formed in those sections of the circular areas, surrounding the irradiation spots, which lie roughly parallel to the polarization direction. The polarization is indicated by the double arrows above the microscope images shown in Figure 3. The relatively large distance from the irradiated spot to the damage zone, the ratio of distance/sample thickness ≈ 1, was in agreement with the results reported in [6,10], where refraction from the crater walls was proposed as a damage formation mechanism. The ratio damage distance/sample thickness ≈ 0.5 was considerably larger than those reported considering other known damage formation mechanism by laser beam; for example, the beam diffraction from the ablated crater. The diffraction of radiation from the opaque zone in the irradiated spot (diffraction from the inverse aperture) in the case of 1 mm thick substrate would produce high intensity ring much closer to the irradiated spot (<50 µm) with the ratio damage distance/sample thickness < 0.05 [11].

### 3.3. Line Damages

**Figure 4:**
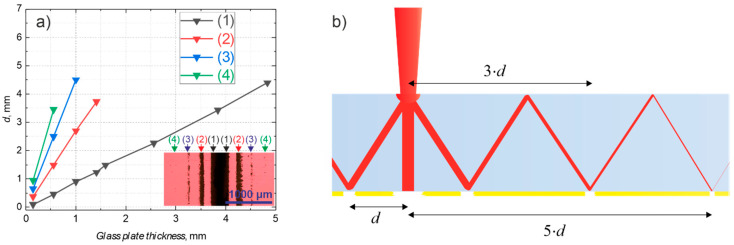
(**a**) distance *d* from the laser scan line to the damage area in the gold coating on various thickness glass plates, (1)–(4) denotes the first, second, etc. damage zones, insert shows damage zones in the coating on 0.15 mm-thick substrate; (**b**) illustration of the formation of high order damage zones by multiple reflections.

The influence of various parameters, such as fluence in the spot size, substrate thickness, orientation, and polarization of the laser beam, to the formation of damage zones when ablating the trench in the back-side of the glass with thin metal coating was investigated. Figure 4a shows a dependence of the distance *d* from the ablation channel to the damaged area on the substrate thickness. It must be noted that in samples containing thin glass substrates (less than 1.6 mm thick), higher-order damage zones also appear and the number of damage zones increases with decreasing substrate thickness. The higher-order damages can be explained by the internal reflection of the radiation, escaping from the ablation channel, on the air/coating/glass and air/glass/interfaces (Figure 4b). The absence of the higher-order damages in thick substrates can be explained by the relatively large optical path length, resulting in the absorption of the escaped radiation. For example, for soda-lime glass, if radiation escapes from the channel at 40° angle to the substrate, in 1 mm thick substrate and is reflected from the glass/coating/air interface back into the substrate it will propagate 2.6 mm, before hitting the glass/coating/air interface again. By using the well-known relation, *I = I*_0_·exp*(−*4*πk/λ·h)* for the intensity attenuation inside material thickness *h*, losses inside the substrates of various thickness can be evaluated. *I*_0_ is initial intensity, *I*—intensity after propagating material thickness *h*, *k* is the extinction coefficient, *λ* is the wavelength. The extinction coefficient for soda-lime at 1064 nm wavelength is *k* = 4.9 × 10^−6^ [14]. After propagation of 2.6 mm distance, the beam intensity is reduced by 14%, compared to the beam reaching the interface the first time. Note that losses due to the transmission through the interface were not accounted. Having the same propagation angle but a 3 mm thick substrate, it will result in a 7.8 mm propagation length and 37% intensity attenuation.

It is worth noting that no higher-order damage zones were observed in the sapphire sample, as shown in Figure 2b. This can also be the result of the relatively low ablation threshold of gold coating directly deposited on soda-lime substrate, due to low adhesion: 0.14 J/cm^2^ and 0.04 J/cm^2^ for 1064 nm and 532 nm wavelengths, respectively.

**Figure 5:**
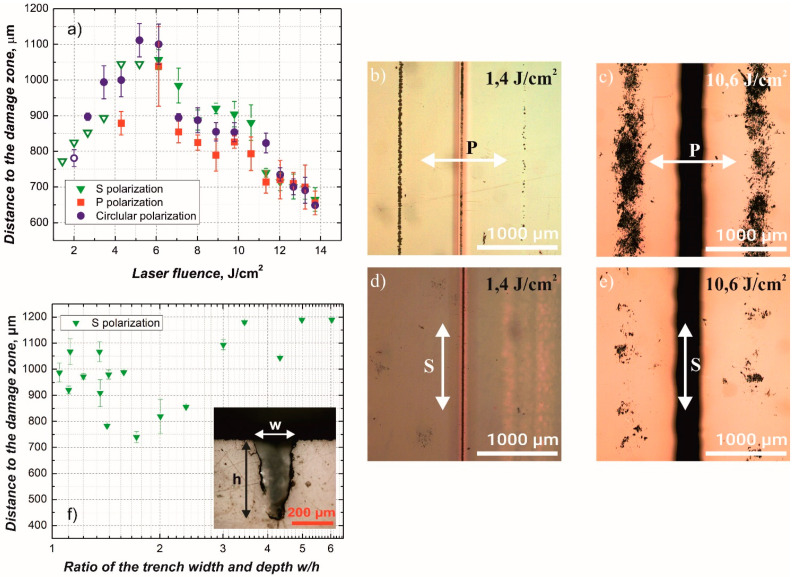
(**a**) Dependence of the distance from the cut line to the damage zone on the laser fluence for S, P and circular polarizations. The distance was measured from the location, corresponding to the center of the ablation channel, to the center of one of the side damage zones. (**b**–**e**) Optical microscope images, showing the coating on the backside of the soda-lime substrate after ablation of the trench, visible in the middle of the image, from the substrate side. (**f**) Dependence of the distance to the damage zone on the ratio between ablated trench width *w* and depth *h*. Laser beam polarization directions are indicated by the arrows. All samples were prepared using 532 nm wavelength, 100 scans, 10,000 pulses per mm.

In Figure 5 experimental results on the variation of polarization state for 532 nm laser wavelength are provided. Figure 5a shows the dependence of the distance from the plane, containing ablated trench, to the damage zone on the laser spot size fluence for S, P, and circular polarization states. The S and P polarizations, in this case, are defined as follows: for S polarization the polarization vector is parallel to the ablated trench and for the P polarization the polarization vector is perpendicular to the ablated trench (vector lies in the plane containing trench cross-section and laser beam).

Orienting the polarization vector parallel to the trench (S polarization), we found a significant reduction of the damage to the backside coating as shown in Figure 5b–e. It can be seen that at low fluence the damage to the coating using S polarization is significantly smaller in comparison to those made using the P polarization. Explanation is a higher transmittance of P polarized radiation through the air/glass interface at ablated trench walls. Our results were in agreement with previous results reported in [6], demonstrating the similar laser induced damage in the back surface of the Schott glass plate, However, in [10] the opposite result (lower damage using S polarization) was reported for fused silica samples. The result was explained by lower reflectance of P polarization at the backside glass/air interface, resulting in the reduction of interference between incident and reflected radiation at this interface, however, our experimental results unambiguously showed lower damage generation using S polarization.

Some alterations in the backside coating could be seen even for S polarization at low fluencies (shown in Figure 5a as hollow symbols). Also, it can be noted that the distance from the trench to the damaged zone depends on the fluence and reaches a peak at about 5.5 J/cm^2^ independent of polarization. This can be related to the change in crater shape with increasing fluence. Figure 5f shows dependence of the distance to the damage zone on the ratio of ablated trench width and depth when using S polarized laser beam. It is evident that the distance to the damage zone changes with the final shape of the ablation crater.

**Figure 6:**
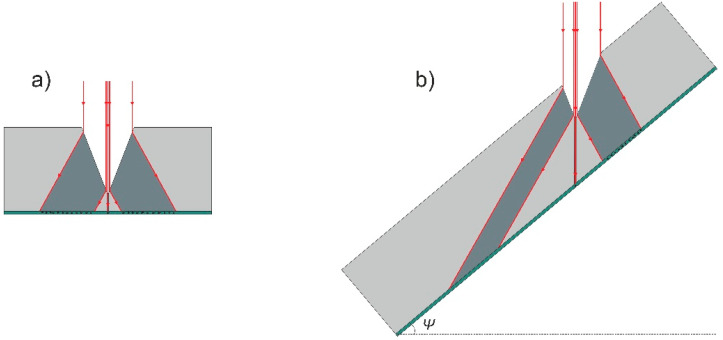
Illustration of damage to the coating, deposited on the backside of laser scribed transparent substrate. (**a**) Schematically shows damage area when a trench is ablated in substrate oriented perpendicularly to the plane in which laser beam is scanned; (**b**) shows damage area when the substrate is oriented at 40° angle to the plane in which laser beam is scanned.

In case the polarization selection is not sufficient to prevent damage in the coating, the “dead zone” in the backside coating along both sides of the scan line can be reduced by orienting a substrate at an angle to the plane, which contains the scanned laser beam. In Figure 6 this is illustrated by comparing two cases: when the substrate is perpendicular to the laser beam (*Ψ* = 0°; Figure 6a) and when the substrate is rotated so that the angle between the substrate and the beam scanning plane is 40° (*Ψ* = 40°; Figure 6b). The crater is shown as a triangle shape due to its simplicity. The rays, showing the laser radiation, propagates to the crater wall and are refracted, according to the Snell’s law. In the case of *Ψ* = 0° zones containing the refracted radiation and reaching the backside coating are formed on both sides of the crater. In the case of *Ψ* = 40° at one side refracted radiation is contained very near the ablation channel, and on the other side propagates into the substrate at a shallow angle, resulting in both losses due to the absorption in the substrate material and in a widening of the zone at which radiation reaches the backside coating, so reducing the probability to damage it.

**Figure 7:**
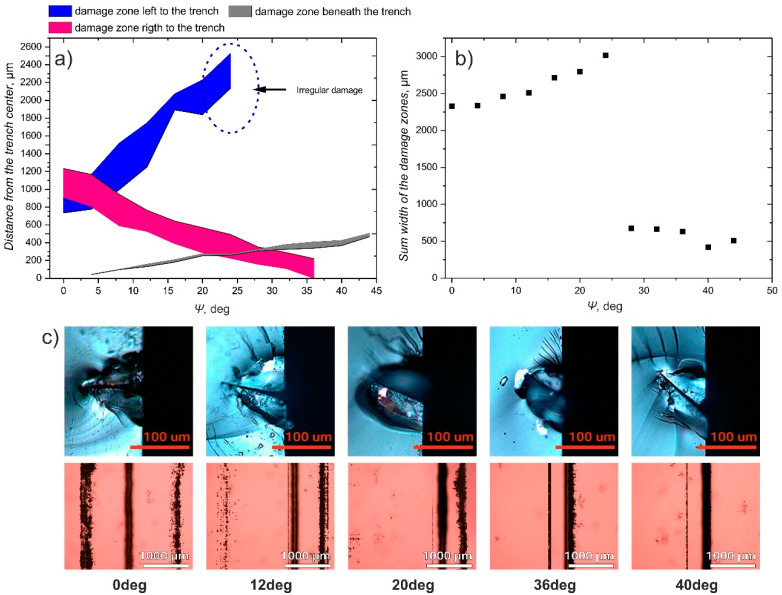
Evaluation of damage zones in the backside coating when changing the angle between the substrate and the beam scanning plane *Ψ*. (**a**) shows the dependence of the distance from the ablation channel to the near and far limits of damage zones formed in both sides of the ablation channel and beneath it (colored bands correspond to the particular damage zone); (**b**) shows the dependence of the sum width of the coating containing damage areas in the vicinity of the ablation channel; (**c**) shows cross-sections of the ablation channels and backside coating beneath the ablation channel for various angles *Ψ*. Laser wavelength 532 nm, polarization direction perpendicular to the scan line (P), 100 scans, 10,000 pulses per mm, 6.1 J/cm^2^.

In Figure 7a, experimentally obtained dependence of the distance from the ablation trench to the damage zones at both sides of the cut line on the angle between the substrate and the beam scanning plane *Ψ* is shown. When *Ψ* = 0°, two damage zones on both sides of the ablation plane are formed at approximately 1000 µm distance from the beam scanning plane. While *Ψ* increases to about 20°, both of these damage zones remain present, although the damage zone on left-hand side becomes weaker, reduced to the isolated islands of damaged or removed coating. Also, all damage zones shift to the left from the cut line resulting in a moderate increase of the sum damage zone width (Figure 7b). At the same time, an increase of the *Ψ* results in the formation of damage area, caused by irradiation transmitted straight through the substrate, not directly beneath the cut line, but at some distance from it due to the substrate orientation. When *Ψ* is further increased the damage zone at the left-hand side becomes even weaker (Figure 7c) and completely disappears at angles equal or larger than 28°, resulting in a sharp drop of the sum damage zone width from 3000 µm to less than 750 µm. When 28° ≤ *Ψ* ≤ 44°, a slight decrease of the sum damage zone width can be observed (Figure 7b), caused by the right damage zone moving beneath the channel, and impeded by the increasing width and distance from the cut line of the damage zone caused by irradiation transmitted straight through the substrate (Figure 7a,c).

**Figure 8:**
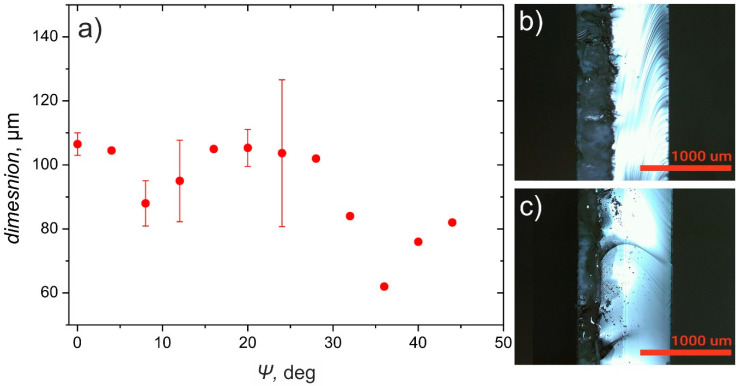
(**a**) Dependency of the ablated crater depth on the sample rotation angle *Ψ*. Laser wavelength 532 nm, polarization direction perpendicular to the scan line (P polarization), 100 scans, 10,000 pulses per mm, 6.1 J/cm^2^; (**b**,**c**) Channels, ablated using *Ψ* = 0° and *Ψ* = 42°, respectively. Other parameters were kept constant: wavelength 532 nm, P polarization, 100 scans, 10,000 pulses per mm, 13.8 J/cm^2^.

It can be concluded that when the selection of the beam polarization is not possible or does not provide sufficiently good results, rotation of a substrate at an angle to the plane which contains the scanned laser beam can be used for reduction of the “dead zone” width 2–3 times. However, it must also be considered that fabrication time required to ablate the channel, deep enough for breaking the substrate along it, in such case would increase. Figure 8 shows the dependence of trench depth on the sample rotation angle *Ψ* for 532 nm wavelength. The trench depth is reduced by 35%, when the substrate rotation angle is changed from 0° to 42°, keeping other parameters constant.

## 4. Performance of Electronic Devices

**Figure 9:**
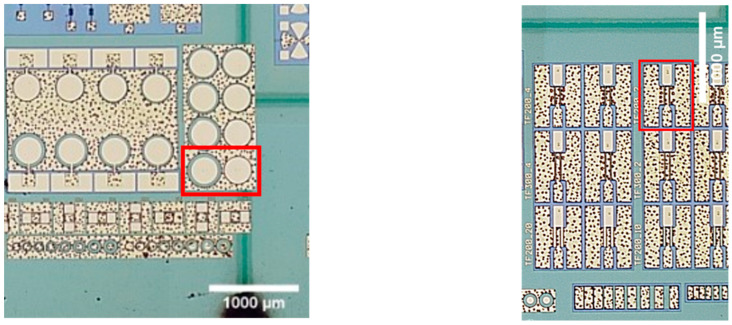
Microscope images of the transparent SiC wafer with AlGaN/GaN heterostructures and electronic devices being in a focus and laser ablated trenches being on the back side. Red color rectangles indicate selected devices for investigations: Schottky barrier diodes (**left**) and High-electron mobility transistor (**right**). Note that the trench line was made directly under one of the diodes. Scale bar is 1000 μm.

**Figure 10:**
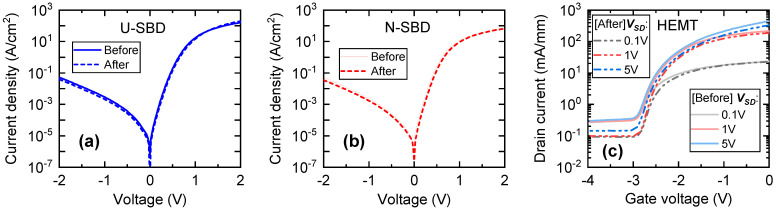
Current-voltage (I-V) characteristics of Schottky barrier diodes (SBDs) (**a**,**b**) and high-electron-mobility transistors (HEMT) (**c**) located under (**a**) and near (approximately 250 μm away) the trenches (**b**,**c**) formed in the back side of the substrate by laser microfabrication. The samples were characterized before and after the trenches were processed on the back side of SiC wafer in a depth of 270 µm.

**Figure 11:**
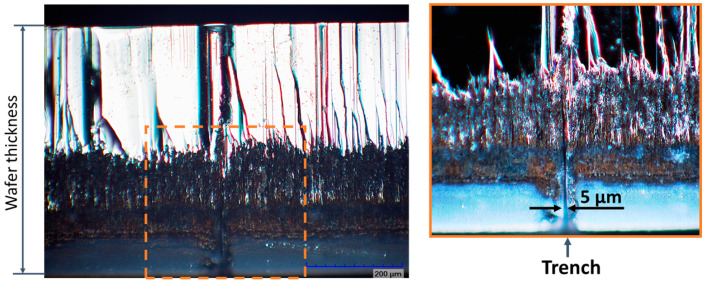
Microscope image of edge of SiC wafer after cleavage along the laser processed trench line (**left**) and zoomed in area (**right**). The depth of the trench formed by laser microfabrication in a perpendicular direction to image plane is of about 270 µm while the width varies from 30 µm down to 5 µm or even smallest values going deeper inside the trench. Scale bar is 200 μm.

Finally, the laser ablation process was optimized and used to dice transparent wafers with AlGaN/GaN heterostructures with various electronic devices. Before laser processing, some of Schottky barrier diodes (SBDs) and High-electron-mobility transistors (HEMTs) were selected for detailed investigation by measuring the I–V characteristics in the EPS150 probe station (Cascade Microtech, Beaverton, OR, USA) equipped with the source-measure-unit SMU Keithley 2400 (Tektronix, Beaverton, OR, USA). Selected devices are indicated by red color rectangle in Figure 9 and their respective electrical characteristics before the processing are shown in Figure 10 by solid lines. Visible trenches appeared after the laser ablation on the back side and are seen in Figure 9 due to the transparency of the substrate. Using the optimized processing parameters, no visible damage to the AlGaN/GaN heterostructures was observed along the ablation lines (see Figure 9). The Schottky barrier diodes (SBDs) used for investigation are indicated by red color rectangle. The depth of the trench was found to be about 270 μm, as it is shown in Figure 11 (right), and the width at the top was found to be about 30 μm with obvious reduction down to a few microns at the maximum depths.

Current-voltage (I-V) measurements were performed on Schottky barrier diodes (SBDs) before and after laser cutting in order to investigate possible laser-induced damage to the active layers of the AlGaN/GaN high electron mobility (HEMT) structure. As seen in Figure 9, two SBDs were chosen in close proximity to the laser cutting lines. One SBD was directly under the cutting line (labelled as U-SBD), while the other selected for the investigation SBD (N-SBD) was approximately 250 μm away from it. The I-V characteristics of the first and second diode after laser dicing are shown in Figure 10a,b by dashed lines, respectively. The transfer characteristics of HEMT were measured in a similar way and results are shown in Figure 10c. All results clearly demonstrate, that the laser micromachining did not affect the I–V characteristics of different electronic devices. Moreover, by fitting the low voltage region of forward current voltage characteristics and using thermionic emission model, the ideality factor, *n*, and Schottky barrier height, *φ*_b_, were extracted. The values of both parameters were found to be about *n* = 1.4 and 0.45 eV, respectively, without a noticeable change due to the laser ablation of trenches on the SiC substrate. A small modification of I–V characteristics of all devices was attributed to different ambient conditions during the measurements, estimating its deviation to be within a range of 2%.

## 5. Conclusions

The influence of various parameters, such as fluence in the spot size, substrate thickness, orientation, and polarization of the laser beam, to the formation of damage zones when ablating the trench in the back-side of the transparent substrate with electronic devices was investigated. The experimental results regarding minimization of the damage to the backside coating were in agreement with the radiation refraction from the ablated crater model described in literature for the back surface damage in a transparent material. We found that the selection of laser beam polarization is not always sufficient to prevent the damage in the coating. The “dead zone” in the backside coating along both sides of the scan line can be reduced up to 2–3 times by using an optimal orientation of substrate, found to be at a 28° angle to the incident plane. However, in this case, the trench depth should be additionally optimized. Proper settings of laser ablation for the selected SiC wafer with back side heterostructures and electronic devices is validated demonstrating laser-based microfabrication and substrate dicing without modification on the electrical characteristics.

## Data Availability

The data that support the findings of this study are available on request from the corresponding author.
